# Occurrence and Survival of Livestock-Associated MRSA in Pig Manure and on Agriculture Fields

**DOI:** 10.3390/antibiotics10040448

**Published:** 2021-04-16

**Authors:** Lӕrke Boye Astrup, Julie Elvekjӕr Hansen, Karl Pedersen

**Affiliations:** 1 National Veterinary Institute, Technical University of Denmark, Kemitorvet, DK-2800 Kongens Lyngby, Denmark; lboast@dtu.dk (L.B.A.); julie.elvekjaer@gmail.com (J.E.H.); 2 Department of Animal Health and Antimicrobial Strategies, National Veterinary Institute, 751 89 Uppsala, Sweden

**Keywords:** LA-MRSA, manure, soil, environment

## Abstract

Livestock-associated methicillin-resistant *Staphylococcus aureus* (LA-MRSA) is widespread in European pig production, and an increasing number of humans attract infections with this bacterium. Although most infections occur in humans with direct livestock contact, an increasing number of infections occur in humans without any established livestock contact. There have been speculations that at least some of these infections may be connected to the exposure of liquid pig manure for example spread as fertilizers. The present study therefore undertook to measure the presence of LA-MRSA in liquid pig manure and on the surface of soils fertilized with liquid manure and investigate the survival of the bacterium in manure. The results showed that LA-MRSA could be detected in 7 out of 20 liquid manure samples and in 12 out of 186 soil samples. However, the bacterium was not more frequently detected in samples collected after compared to before the spreading of liquid manure on the fields, thus suggesting that other sources of LA-MRSA on agriculture fields likely exist. The decimation time in liquid manure was >32 days at 5 °C in vitro but decreased with increasing temperature. Based on these results, liquid manure does not appear to be an important risk factor for human exposure to LA-MRSA.

## 1. Introduction

Several studies have investigated the occurrence of antibiotic residues and antibiotic-resistant bacteria in manure from farms, particularly in pig manure, and it has been shown that both are common in manure [1]. Furthermore, a German study demonstrated that liquid pig manure generally contained more bacteria that were resistant to several antibiotics than urban sewage sludge [2]. Similar results were also demonstrated in a study conducted in Korea [3]. While previous investigations foremost have focused on *Escherichia coli* or enterococci, or applied metagenomics, few studies have so far to our knowledge investigated the occurrence of methicillin-resistant *Staphylococcus aureus* (MRSA) in pig manure, and none have addressed the survival of this bacterium in manure.

MRSA was first described in the 1960s in humans but has since then become widespread in the world in both humans and animals [4]. Various clonal lineages of MRSA are recognized, some mostly connected to humans, while others are more connected to animals—so-called livestock-associated MRSA (LA-MRSA). One such strain of LA-MRSA is CC398, which was first described in 2005 [5] and has since then spread extensively through European animal production [6,7,8]. In Denmark, LA-MRSA CC398 is widespread and is found in most pig herds [9,10] and, although to a lesser extent, also among other animal species [9,11,12]. CC398 is readily transferred to humans principally via direct animal contact, and consequently, farm workers, veterinarians, and slaughterhouse workers have been shown to carry LA-MRSA more often than the rest of society [13,14,15]. Furthermore, infected or contaminated persons can also transmit LA-MRSA to other members of their household [16]. This is also reflected in the fact that LA-MRSA is considerably more widespread in rural areas compared to urban areas [17].

While most human LA-MRSA carriers have direct or indirect contact with livestock, an increasing number of persons living in rural areas have been found to be carriers or infected with this bacterium without any recognized livestock contact. The transmission routes for these people are unknown although there are indications that some might have had some unrecognized, indirect livestock contact [17,18]. It has also been suggested that LA-MRSA could spread to the surrounding human population from farms via ventilation systems, flies escaping barns, or contaminated manure spread on farmland as fertilizers, but studies are scarce and nonconclusive [18,19]. New studies suggest that LA-MRSA can survive on house flies for at least 72 h [19]. It remains uncertain whether these sources contribute to the spreading of LA-MRSA into society. However, proximity to fields applied with pig manure was shown to be an independent risk factor of MRSA infections in humans in a study from the US [20]. While a limited number of studies have investigated the occurrence of MRSA in manure, it is well known that various MRSA strains can be detected in wastewater treatment plants [21].

The present investigation aimed to elucidate (i) whether and to which extent LA-MRSA occurs in liquid pig manure, (ii) whether it can be detected on soil surfaces before and after spreading of liquid pig manure, and (iii) for how long LA-MRSA survives in liquid pig manure and the effect of temperature on the survival. This type of information is much needed to perform risk analyses for human exposure of LA-MRSA from liquid pig manure used as fertilizer.

## 2. Results

An overview of sample types analyzed with in vitro studies is given in Supplementary Table S1.

### 2.1. Survival of LA-MRSA in Natural Positive Manure

LA-MRSA in natural positive pig manure had a decimation time of at least 32 days at 5 °C and almost 15 days at 15 °C. At 37 °C, LA-MRSA was undetectable already after 24 h. The natural positive manure stayed LA-MRSA positive by enrichment throughout the study time of one month. The natural load of LA-MRSA in natural positive pig manure was 20–115 CFU/mL.

### 2.2. Survival of LA-MRSA in Spiked Manure

LA-MRSA in spiked pig manure had a decimation time of up to 30.5 days at 5 °C. The survival of *spa*-type t011 and t034 did not differ but was markedly temperature dependent (Table 1).

**Figure 1:**
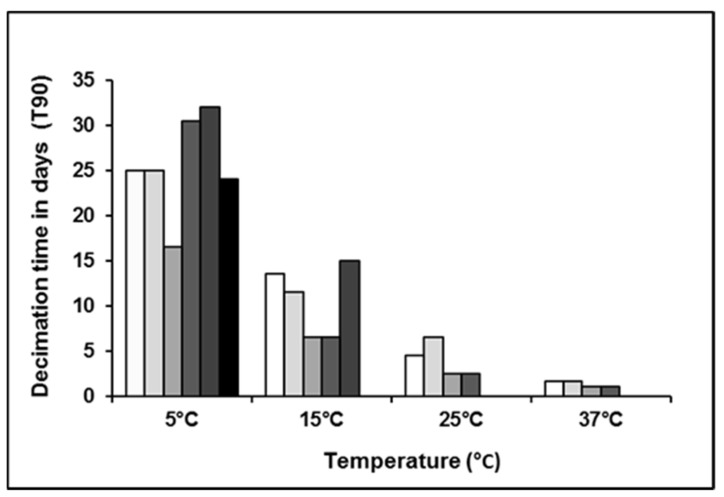
Decimation times of various initial LA-MRSA loads at different temperatures.

In addition to temperature, the initial load of LA-MRSA also influenced the decimation time, with the high spiking dose having a longer decimation time than the low spiking dose (Figure 1). White = t011 low load, light gray = t034 low load, gray = t011 high load, semidark gray = t034 high load, dark gray = natural sample 100 CFU/mL, black = natural sample = 20 CFU/mL.

**Figure 2:**
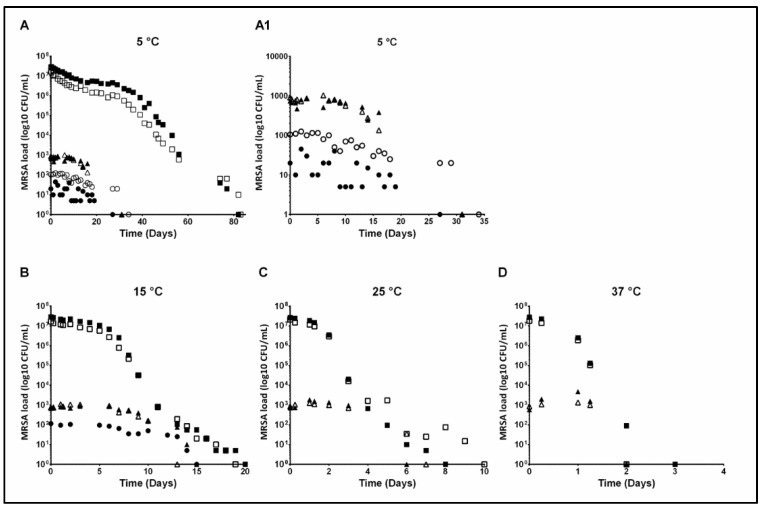
Survival curves of LA-MRSA in natural positive samples and spiked samples at four different temperatures; (**A**): 5 °C; (**A1**): 5 °C, a close-up of low levels from (**A**); (**B**): 15 °C; (**C**): 25 °C; (**D**): 37 °C.

The survival curves for LA-MRSA in both natural positive and spiked manure samples are shown in Figure 2. Closed square = t034 107 CFU/mL, open square = t011 107 CFU/mL, closed triangle = t011 102 CFU/mL, open triangle = t034 102 CFU/mL. Figure 2A is at 5 °C, closed circle = natural pos. sample 20 CFU/mL, open circle = natural sample 100 CFU/mL. Figure 2A1, 5 °C, is a close-up of low levels from Figure 2A. Figure 2B is 15 °C, circle closed = natural sample 100 CFU/mL. Figure 2C is 25 °C and Figure 2D 37 °C. With a high spiking level the decay of LA-MRSA, until decimation time, at 5 °C could be explained by exponential regression, whereas the decay at 15, 25, and 37 °C could be explained by linear regression. The decay in samples with low spiking, irrespective of temperature, was less predictable and could not be described by either linear or exponential regression. At 5 °C LA-MRSA was stable until Day 7, whereas an initial slight increase in LA-MRSA was observed at 15, 25, and 37 °C, before a continuous decay of LA-MRSA could be detected (Figure 2).

### 2.3. Screening of LA-MRSA in Manure Storage Tank Samples from Farms

The screening of manure showed that 37% (7/19) of the screened tanks were positive for LA-MRSA. All positive samples were positive by direct plating on selective MRSA 2 agar with a range of 5–280 CFU/g (mean = 83 CFU/g).

### 2.4. LA-MRSA in Boot Sock Samples from Fields before and after Fertilization

Five fields were identified as LA-MRSA positive in ≥1 sample before fertilization, and three fields were found LA-MRSA positive in ≥1 sample after fertilization (Table 2).

Three fields went from a LA-MRSA-positive status before to LA-MRSA-negative status after fertilization, whereas two fields were LA-MRSA positive before as well as after fertilization. However, none of the farms with a LA-MRSA-positive field status had detectable LA-MRSA in the manure tanks. A single field was found LA-MRSA negative prior to fertilization and positive for LA-MRSA after, with that farm also negative for LA-MRSA in the manure sample. In positive samplings, between one and three out of the five pairs of sock samples were found positive (Table 2). There was no statistical difference (*p* < 0.05) between the number of farms positive for LA-MRSA in soil samples, nor the total number of boot sock samples positive before and after the spreading of manure.

## 3. Discussion

To our knowledge, no studies have so far addressed the presence of LA-MRSA in liquid manure tanks and the contribution of this liquid manure to the contamination of agricultural fields with LA-MRSA, nor have studies been carried on the survival and decimation times of LA-MRSA in manure as a function of temperature. This investigation was carried out to elucidate these matters.

Our results show that LA-MRSA can survive for at least 32 days at 5 °C and 15 days at 15 °C in liquid pig manure (Table 1). The investigated temperatures were chosen to represent expected low and high temperatures in Danish manure tanks. The decimation time in the manure spiked with 2.87 × 10^7^ CFU/mL LA-MRSA was at least 6.5 days at 15 °C. A previous study by Munch et al. [22] reported similar decimation times for *Staphylococcus aureus* in liquid manure and also a marked dependence on temperature. In addition, these authors reported a marked difference between aerated and nonaerated liquid manure, i.e., a mean decimation time of 0.7 weeks at 18–20 °C and 2.6 weeks at 6–9 °C in aerated liquid manure but 0.9 and 7.1 weeks, respectively, in nonaerated liquid manure. A decimation time up to 17 weeks was reported [21]. As such, our results on the survival time and decimation time for the two different LA-MRSA strains seem equal to those of methicillin-susceptible *S. aureus*. Additionally, Levin-Edens et al. [23], who compared the survival of MRSA strains in water in microcosms at 13 °C and 20 °C, found longer survival times of MRSA at the lower temperature. In addition, these authors found longer time survival in marine water compared to freshwater.

Although the present survival studies were carried out in laboratory set-ups, we are confident that the results are applicable to field conditions. Regarding the risk of the environmental dissemination of LA-MRSA through manure, our results indicate that LA-MRSA is indeed able to survive during normal liquid manure storage conditions. Furthermore, the duration of LA-MRSA survival that we observed in the in vitro experiments is sufficient to account for the risk that the LA-MRSA can pass through the storage tank and reach the fertilized fields in a viable state during seasons of manure application. Storage of swine slurry with retention time before application on fields might decrease the number of viable LA-MRSA passed on to the environment. It must be anticipated that LA-MRSA is continuously led to the storage tank from the barns, i.e., the detected LA-MRSA can, in principle, be between 0 days and up to several months old. This may have an impact on the viability of the bacterium after application on the fields. Investigations on *S. aureus* have demonstrated that the bacterium may enter a viable but nonculturable (VBNC) state when stored in seawater. At 4 °C, the bacterium was able to survive for at least 120 days under these conditions [24]. Such adaptations to challenging environments might take place in MRSA in manure, too. Studies on the survival of LA-MRSA in dust in pig farms indicated a half-life of 5 days at room temperature, corresponding to a decimation time of approximately 17 days [25], which is somewhat longer than the decimation time (2.5–6.5 days, Table 2) we found in liquid manure at 25 °C.

The survival time of LA-MRSA after application of the manure on the fields is not known and needs to be further investigated. It must be anticipated that the degradation of the bacterium will depend on several physical and chemical factors, such as seasonal ambient temperature, precipitation, UV irradiation from sunlight, chemical and nutritional composition of the soil, concurrent antagonistic microflora, etc. Likewise, liquid manure is now more often deposited some centimeters below the soil surface to reduce smell disturbances for the neighbors and reduce nitrogen loss. This is likely to also impact the deposition of LA-MRSA on the soil surface and thereby the risk to human exposure.

People living in rural, livestock-dense areas are significantly more often positive for LA-MRSA than people living in urban areas [13,17,26], and there is a clear association between the likelihood of being LA-MRSA positive and the intensity and duration of livestock contact [27,28]. Although the majority of LA-MRSA-positive persons have direct (farmworkers, slaughterhouse workers, livestock transporters, veterinarians) or indirect livestock contact (household members, visitors to farms), there are still persons with no animal contact who become LA-MRSA positive. Casey et al. [20] found that the risks of community-acquired MRSA (CA-MRSA), healthcare-associated MRSA (HA-MRSA), and soft-tissue infections (unknown etiology) all increased significantly with higher swine manure exposure. A similar but less strong association was found with cattle manure [20]. The relation was not affected by distance to crop fields as such but solely by exposure to manure-treated fields [20]. Additionally, high-density swine production was an independent risk factor for both HA-MRSA, CA-MRSA, and soft-tissue infections [20]. Consequently, public concern has been raised, in particular from neighbors to pig farms, as to the risk associated with manure. In the present study, we therefore set out to measure the presence of LA-MRSA in liquid pig manure as well as on fields where manure had been spread, and we studied the survival of LA-MRSA in liquid pig manure.

The fact that LA-MRSA was not found in all manure samples, despite that all farms were LA-MRSA positive among the pigs, together with the observation that LA-MRSA was detected in some soil samples before the spreading of manure on some locations, and not after spreading of manure on other locations, suggests that manure is probably not an important source of LA-MRSA in the environment and consequently not an important source for human exposure. Likewise, the fact that we found more sock samples from soil positive before the spreading of manure than after suggests that other sources of LA-MRSA in the environment are more important. A reason why soil samples from a field can be positive before the spreading of manure but negative after (Table 1) may be that the LA-MRSA on the fields before the spreading of manure is the result of the precipitation of LA-MRSA from outlet air from the stables downwind. They may have disappeared due to sun irradiation, rainfall, or other factors before the spreading of manure. Additionally, the concentrations of LA-MRSA in the manure may have been so low that levels after spreading on the soil have gone below the detection limit. However, this needs to be further investigated.

It is well known that LA-MRSA is present in both air and dust in pig farms and constitutes a major exposure risk to farmworkers [29,30]. Studies by Friese et al. [31] and Schulz et al. [32] of samples from air and soil collected outside pig and poultry farms showed that LA-MRSA could be detected in air samples downwind but not upwind from the farms and on soil at least 300 m downwind but much less upwind. Gibbs et al. [33] also found significantly more resistant bacteria in the air plume downwind from a pig farm than upwind. Schulz et al. [32] found more samples positive during the summer period, which makes sense since more ventilation is needed during the summer season and consequently gives more exhaust air outlet from the farms.

Concentrations of LA-MRSA may be high inside pig farms, especially in the weaning sections. Schulz et al. [32] found 6–3.619 (median = 151) CFU/m^3^, which corresponds well with figures obtained by Hansen [34], who found 0–8.650 CFU/m^3^, and Bӕkbo et al. [35], who found an average of 16–40 CFU/m^3^ in a weaning-to-slaughter farm. The ventilation systems on a pig farm may exchange thousands of cubic meters of air per day, which means that exhaust air lets out tremendous numbers of LA-MRSA and other bacteria to the surroundings, where they may gradually sediment on soil surfaces. This may explain why we found some of our soil samples positive for LA-MRSA before the spreading of manure and that the number of positive samples did not increase after spreading.

In conclusion, we found that liquid pig manure may contain LA-MRSA, although in modest concentrations. When spread on agricultural soil surfaces as fertilizer, LA-MRSA may be diluted to undetectable levels. Although liquid manure must be considered a source of contamination of the environment with LA-MRSA, we believe it must be considered a low risk for humans. During in vitro experiments, we demonstrated that the bacterium can survive for several weeks at low temperatures.

LA-MRSA is only one concern with respect to antimicrobial-resistant bacteria, antimicrobial resistance genes, and antimicrobial residues in manure. It is well known that manure contains antimicrobial-resistant bacteria as well as residues [1,2,3]. How these bacteria and residues affect the microbial populations indigenous to soil is less well studied, and it is likewise unknown for how long they persist and to which extent resistance genes are transmitted between bacteria in soil. Further studies seem needed to investigate the survival of LA-MRSA in and on soil and whether the resistance mechanism can be transferred to soil bacteria.

## 4. Materials and Methods

The study included laboratory experiments and field studies. In the laboratory, the decimation times of LA-MRSA in both natural LA-MRSA CC398-positive pig manure and LA-MRSA CC398-spiked pig manure at different temperatures was examined. In the field study, the LA-MRSA status of pig manure storage tanks and of fields before and after fertilization with manure from the same storage tanks was investigated. The field study was designed to reflect the LA-MRSA CC398 dissemination from a fertilized field to a human pedestrian taking 50 steps on the fertilized field.

### 4.1. LA-MRSA Survival in Liquid Pig Manure

#### 4.1.1. Study Design

Positive manure was defined as unspiked manure found positive for LA-MRSA either by direct plating or after an enrichment step, whereas negative manure was defined as unspiked manure not found positive for LA-MRSA. The survival of LA-MRSA in both positive liquid pig manure and in spiked manure was analyzed. The survival was analyzed both as the decimation time and as the absolute survival time. Negative pig manure samples were included as negative controls. All samples (positive, spiked, and negative controls) were kept in sealed plastic containers in the dark and without aeration or rotation. Each sample type was prepared in 3 or 4 series in parallel, with one from each series kept at temperatures of 5, 15, 25, and 37 °C (see Supplementary Table S1 for details). For every sample at each temperature, a count of colony-forming units (CFU) was carried out every 24 h until the samples were negative or up to a maximum of 80 days.

#### 4.1.2. LA-MRSA Strains

For spiking, two strains of LA-MRSA CC398 were examined in parallel. Both strains had previously been obtained from conventional Danish pig farms. Strain 1 belonged to *spa*-type t034, and Strain 2 belonged to *spa*-type t011 (spiking Strains 1 and 2, respectively).

#### 4.1.3. CFU Counts and Estimation of Decimation Rates

A 100 µL volume of the samples was used for serial ten-fold dilutions in phosphate-buffered saline (PBS). From each dilution, 100 µL was plated in duplicate on selective Brilliance^TM^ MRSA 2 plates (Oxoid, Basingstoke, UK). The plates were incubated at 37 °C for 18–24 h, and positive colonies, according to the manufacturer’s instructions, were then counted on all plates. CFU counts were then converted to CFU/g manure according to the criteria specified by ISO 4833 [36].

The CFU/g were plotted for each day, and these plots were used to calculate the decimation time. When the CFU/g over time depicted a linear regression, the decimation time (T_90_) was calculated as (T_90_) = (*y* − *b*)/*a*. When the CFU/g over time depicted an exponential regression, the decimation time (T_90_) was calculated as (T_90_) = (*Ln*(*y*/*b*))/*a*, where *y* is the CFU/g at spiking time 0, *b* is the intercept of the line on the *y*-axis, and *a* is the slope of the line.

#### 4.1.4. Spiking Dosages

The two spiking strains were each inoculated in swine manure at a low (~10^3^ CFU/mL) or high (~10^7^ CFU/mL) dosage. At start, time 0, all spiked samples were plated in parallel onto blood agar plates containing 5% calf blood and on MRSA 2 agar, which was done to confirm the dose and to confirm that growth on the MRSA 2 plates correlated to growth on the blood agar plates.

### 4.2. LA-MRSA in Liquid Manure Storage Tank Samples and Agriculture Fields

At 19 conventional pig farms, fields before and after fertilization and manure storage tanks were screened between March and April 2017.

Storage tank samples were taken once from each farm 2–14 days before fertilization of the fields, and the samples were taken directly from the tank at a depth of at least 1 m below the surface to avoid the floating top layer of manure. All samples were stored at 5 °C and processed within 72 h. To increase detection sensitivity, manure was, in addition to CFU plating, also cultured using broth enrichment, with 1 mL of sample inoculated in 9 mL of Mueller–Hinton broth supplemented with 6.5% NaCl (MHB+), and serial ten-fold dilutions were prepared. Following 18–24 h of incubation at 37 °C with rotation, a 10 µL loopful of each enriched culture was plated on Brilliance MRSA 2 agar and assessed for MRSA.

Agriculture fields were sampled using boot sock samples (Sodibox, Névez, France) as described in Skov et al. [37]. Samples were taken from a single field on each farm within 2 weeks before and 3–8 days after fertilization (“field samples before” and “field samples after,” respectively). A total of 50 steps per field were taken by five sets of sock samples covering a field area of approximately 200 m^2^. For each sock sample, five steps were taken into the field and back out again, beginning at the verge of the field and walking straight into the field and back. The left and right socks were then pooled in a sterile plastic bag producing one sock sample representing 10 steps of field walking within the outermost 5 m of the field area. Then, ten steps were taken alongside the verge of the field, where new protective plastic overdrafts and a pair of socks were put on, and the ten-step sampling was repeated. Thus, five samples were processed from each field per sampling, each consisting of one pair of socks.

Boot sock samples were kept at 5 °C and analyzed within 72 h after collection. A volume of 100 mL MHB+ was added to each sock sample and stomached for 2 min and 30 s followed by incubation at 18–24 h at 37 °C. After incubation, the samples were plated on selective MRSA 2 agar, and CFU counts were performed as previously described. In addition, presumptive MRSA colonies were subcultured on blood agar plates from where colony material was identified as MRSA by PCR detection of the *mecA* and *nuc* genes [38]. Differences between the number of positive farms and the total number of positive sock samples before and after deposition of manure were calculated using Fisher’s exact test with *p* > 0.05 considered statistically significant.

## 5. Conclusions

LA-MRSA can be found in liquid pig manure, although in modest concentrations. The bacterium can survive for extended periods of time, several weeks, in liquid manure, in particular at lower temperatures, and will therefore be spread on fields with the manure. We found some fields positive for LA-MRSA even before the spreading of manure; however, we did not find a higher proportion of fields positive after than before the spreading. Liquid pig manure must therefore be considered a source of LA-MRSA in the environment, but our results suggest that other sources are more important.

## Figures and Tables

**Table 1:** Overview of T90 at different temperatures and initial livestock-associated methicillin-resistant *Staphylococcus aureus* (LA-MRSA) loads.

Sample ID	Temperature (°C)	Initial Load CFU/mL	T90 (Days)
t011 low load	5	7.40 × 10^2^	25
t034 low load	5	9.10 × 10^2^	25
t011 high load	5	1.72 × 10^7^	16.5
t034 high load	5	2.83 × 10^7^	30.5
Natural positive	5	2.00 × 10^1^	24
Natural positive	5	1.05 × 10^2^	32
t011 low load	15	6.75 × 10^2^	13.5
t034 low load	15	7.55 × 10^2^	11.5
t011 high load	15	1.67 × 10^7^	6.5
t034 high load	15	2.87 × 10^7^	6.5
Natural positive	15	1.15 × 10^2^	15
t011 low load	25	7.30 × 10^2^	4.5
t034 low load	25	8.55 × 10^2^	6.5
t011 high load	25	2.13 × 10^7^	2.5
t034 high load	25	2.62 × 10^7^	2.5
t011 low load	37	5.85 × 10^2^	1.6
t034 low load	37	8.45 × 10^2^	1.6
t011 high load	37	1.82 × 10^7^	1.1
t034 high load	37	2.76 × 10^7^	1.1

**Table 2:** MRSA status of manure and sock samples.

Farm ID *n* = 19	Manure Status *	Sock Sample before Fertilization		Sock Sample after Fertilization	
		Status	Positive/*n* Samples	Status	Positive/*n* Samples
1	−	+	3/5	−	0/5
2	−	−	0/1	−	0/5
3	−	+	1/2	+	1/5
4	−	−	0/5	+	2/5
5	+	+	1/3	−	0/3
6	−	−	0/1	−	0/5
8	−	−	0/4	−	0/5
9	−	−	0/5	−	0/5
10	−	−	0/5	−	0/5
11	−	−	0/4	−	0/3
12	−	+	1/5	−	0/5
13	+	−	0/5	−	0/5
14	+	−	0/5	−	0/5
15	+	−	0/5	NA **	NA
16	+	−	0/5	−	0/5
17	+	−	0/5	−	0/5
18	+	−	0/5	−	0/5
19	−	+	1/5	+	2/5
20	−	−	0/5	−	0/5
Total pos	7/19	5/19	7/80	3/18	5/86
% pos	37%	26%	9%	17%	6%

* + indicates positive and − indicates negative. ** NA indicates that samples from this field were not obtained.

## Data Availability

The datasets analyzed during the current study are available from the corresponding author on reasonable request.

## Supplementary Materials

The following are available online at https://www.mdpi.com/article/10.3390/antibiotics10040448/s1, Table S1: Overview of sample types at different temperatures analyzed in the in vitro survival studies.

## References

[1] Rasschaert G., Van Elst D., Colson L., Herman L., Cardoso de Carvalho Ferreira H., Dewulf J., Decrop J., Meirlaen J., Heyndrickx M., Daeseleire E. (2020). Antibiotic residues and antibiotic-resistant bacteria in pig slurry used to fertilize agricultural fields. Antibiotics.

[2] Hölzel C.S., Schwaiger K., Harms K., Küchenhof H., Kunz A., Meyer K., Müller C., Bauer J. (2010). Sewage sludge and liquid pig manure as possible sources of antibiotic resistant bacteria. Environ. Res..

[3] Lee J., Shin S.G., Jang H.M., Kim Y.B., Lee Y., Kim Y.M. (2017). Characterization of antibiotic resistance genes in representative organic solid wastes: Food waste-recycling wastewater, manure, and sewage sludge. Sci. Total Environ..

[4] Lee A.S., de Lencastre H., Garau J., Kluytmans J., Malhotra-Kumar S., Peschel A., Harbarth S. (2018). Methicillin-resistant *Staphylococcus aureus*. Nat. Rev. Dis. Primers.

[5] Voss A., Loeffen F., Bakker J., Klaassen C., Wulf M. (2005). Methicillin-resistant *Staphylococcus aureus* in pig farming. Emerg. Infect. Dis..

[6] European Food Safety Agency (EFSA) (2009). Analysis of the baseline survey on the prevalence of methicillin-resistant *Staphylococcus aureus* (MRSA) in holdings with breeding pigs, in the EU, 2008—Part A: MRSA prevalence estimates. EFSA J..

[7] European Medicines Agency (EMA) (2009). Reflection Paper on MRSA in Food Producing and Companion Animals in the European Union: Epidemiology and Control Options for Human and Animal Health. https://www.ema.europa.eu/en/documents/scientific-guideline/reflection-paper-mrsa-food-producing-companion-animals-european-union-epidemiology-control-options_en.pdf.

[8] Lekkerkerk W.S.N., van de Sande-Bruinsma N., van der Sande M.A.B., Tjon-A-Tsien A., Groenheide A., Haenen A., Timen A., van den Broek P.J., van Wamel W.J.B., de Neeling A.J. (2012). Emergence of MRSA of unknown origin in the Netherlands. Clin. Microbiol. Infect..

[9] DANMAP-2016 (2017). Use of Antimicrobial Agents and Occurrence of Antimicrobial Resistance in Bacteria from Food Animals, Food and Humans in Denmark.

[10] Danish Veterinary and Food Administration (2017). Resultaterne af Screening for Husdyr-MRSA i Svin i 2016. https://www.foedevarestyrelsen.dk/Nyheder/Aktuelt/Documents/MRSA%20ekspertgruppe%20-%20resultatene%20forekomst%20af%20husdyr-MRSA%20i%20svin%202016.pdf.

[11] Hansen J.E., Larsen A.R., Skov R.L., Chriél M., Larsen G., Angen Ø., Larsen J., Lassen D.C.K., Pedersen K. (2017). Livestock-associated methicillin-resistant *Staphylococcus aureus* is widespread in farmed mink (*Neovison vison*). Vet. Microbiol..

[12] Hansen J.E., Ronco T., Stegger M., Sieber R., Fertner M.E., Martin H.L., Farre M., Toft N., Larsen A.R., Pedersen K. (2019). MRSA CC398 in dairy cattle and veal calf farms indicates spillover from pig production. Front. Microbiol..

[13] Bisdorff B., Scholhölter J.L., Claussen K., Pulz M., Novak D., Radon K. (2012). MRSA-ST398 in livestock farmers and neighbouring residents in a rural area in Germany. Epidemiol. Infect..

[14] Cuny C., Wieler L., Witte W. (2015). Livestock-associated MRSA: The impact on humans. Antibiotics.

[15] Mulders M.N., Haenen A.P.J., Geenen P.L., Vesseur P.C., Poldervaart E.S., Bosch T., Huijsdens X.W., Hengevelt P.D., Dam-Deisz W.D.C., Graat E.A.M. (2010). Prevalence of livestock-associated MRSA in broiler flocks and risk factors for slaughterhouse personnel in The Netherlands. Epidemiol. Infect..

[16] Dahms C., Hübner N.-O., Cuny C., Kramer A. (2014). Occurrence of methicillin-resistant *Staphylococcus aureus* in farm workers and the livestock environment in Mecklenburg-Western Pomerania, Germany. Acta Vet. Scand..

[17] Danish Veterinary and Food Administration (2017). MRSA Risiko og Håndtering. Rapport ved MRSA-Ekspertgruppen..

[18] Toft N., Larsen A.R., Pedersen K., Koch A. (2019). OHLAM-Projektet, en One-Health Forskningsindsats om Husdyr-MRSA Hos Dyr Og Mennesker.

[19] Stelder J.J., Kjær L.J., Jensen L.B., Boklund A.E., Denwood M., Carlsen M., Bødker R. (2021). Livestock-associated MRSA survival on house flies (*Musca domestica*) and stable flies (*Stomoxys calcitrans*) after removal from a Danish pig farm. Nat. Sci. Rep..

[20] Casey J.A., Curriero F.C., Cosgrove S.E., Nachman K.E., Schwartz B.S. (2013). High-density livestock operations, crop field application of manure, and risk of community-associated methicillin-resistant *Staphylococcus aureus* infection in Pennsylvania. JAMA Intern. Med..

[21] Börjesson S., Mernelius S., Matussek A., Lindgren P.-E. (2009). A seasonal study of the *mecA* gene and *Staphylococcus aureus* including methicillin-resistant *S. aureus* in a municipal wastewater treatment plant. Water Res..

[22] Munch B., Errebo Larsen H., Aalbӕk B. (1987). Experimental studies on the survival of pathogenic and indicator bacteria in aerated and non-aerated cattle and pig slurry. Biol. Wastes.

[23] Levin-Edens E., Bonilla N., Scott Meschke J., Roberts M.C. (2011). Survival of environmental and clinical strains of methicillin-resistant *Staphylococcus aureus* [MRSA] in marine and fresh waters. Water Res..

[24] Masmoudi S., Denis M., Maalej S. (2010). Inactivation of the gene *katA* or *sodA* affects the transient entry into the viable but non-culturable response of *Staphylococcus aureus* in natural seawater at low temperature. Mar. Pollut. Bull..

[25] Feld L., Bay H., Angen Ø., Larsen A.R., Madsen A.M. (2018). Survival of LA-MRSA in dust from swine farms. Ann. Work Expo. Health.

[26] Feingold B.J., Silbergeld E.K., Curriero F.C., van Cleef B.A.G.L., Heck M.E.O.C., Kluytmans J.A.J.W. (2012). Livestock density as risk factor for livestock-associated methicillin-resistant *Staphylococcus aureus*, the Netherlands. Emerg. Infect. Dis..

[27] Graveland H., Wagenaar J.A., Bergs K., Heesterbeek H., Heederik D. (2011). Persistence of livestock associated MRSA CC398 in humans is dependent on intensity of animal contact. PLoS ONE.

[28] Van Cleef B.A.G.L., Graveland H., Haenen A.P.J., van de Giessen A.W., Heederik D., Wagenaar J.A., Kluytmans J.A.J.W. (2011). Persistence of livestock-associated methicillin-resistant *Staphylococcus aureus* in field workers after short-term occupational exposure to pigs and veal calves. J. Clin. Microbiol..

[29] Bos M.E.H., Verstappen K.M., van Cleef B.A.G.L., Dohmen W., Dorado-Garcia A., Graveland H., Duim B., Wagenaar J.A., Kluytmans J.A.J.W., Heederik D.J.J. (2016). Transmission through air as a possible route of exposure for MRSA. J. Expo. Sci. Environ. Epidemiol..

[30] Friese A., Schulz J., Hoehle L., Fetsch A., Tenhagen B.A., Hartung J., Roesler U. (2012). Occurrence of MRSA in air and housing environment of pig barns. Vet. Microbiol..

[31] Friese A., Schulz J., Zimmermann K., Tenhagen B.-A., Fetsch A., Hartung J., Rösler U. (2013). Occurrence of livestock-associated methicillin-resistant *Staphylococcus aureus* in turkey and broiler barns and contamination of air and soil surfaces in their vicinity. Appl. Environ. Microbiol..

[32] Schulz J., Friese A., Klees S., Tenhagen B.A., Fetsch A., Rösler U., Hartung J. (2012). Longitudinal study of the contamination of air and of soil surfaces in the vicinity of pig barns by livestock-associated methicillin-resistant *Staphylococcus aureus*. Appl. Environ. Microbiol..

[33] Gibbs S.G., Green C.F., Tarwater P.M., Mota L.C., Mena K.D., Scarpino P.V. (2006). Isolation of antibiotic-resistant bacteria from the air plume downwind of a swine confined or concentrated animal feeding operation. Environ. Health Perspect..

[34] Hansen J.E. (2018). Methicillin-Resistant *Staphylococcus Aureus* in Danish Production Animals. Ph.D. Thesis.

[35] Bӕkbo P., Sommer H.M., Pedersen K., Nielsen M.W., Fertner M.E., Espinosa-Gongora C. Forsøg med Nedsӕttelse af Forekomsten af MRSA i Grise og i Staldmiljø. https://svineproduktion.dk/publikationer/kilder/lu_medd/2019/1185.

[36] ISO 4833-2:2013 (2013). Microbiology of the Food Chain—Horizontal Method for the Enumeration of Microorganisms—Part 2: Colony Count at 30 °C by the Surface Plating Technique.

[37] Skov M.N., Carstensen B., Tornøe N., Madsen M. (1999). Evaluation of sampling methods for the detection of *Salmonella* in broiler flocks. J. Appl. Microbiol..

[38] Maes N., Magdalena J., Rottiers S., De Gheldre Y., Struelens M.J. (2002). Evaluation of a triplex PCR assay to discriminate *Staphylococcus aureus* from coagulase-negative staphylococci and determine methicillin resistance from blood cultures. J. Clin. Microbiol..

