# Supplementary material for: Occurrence and Survival of Livestock-Associated MRSA in Pig Manure and on Agriculture Fields
Source: Antibiotics (Basel). 2021 Apr 16;10(4):448. doi: 10.3390/antibiotics10040448 (PMC8071562; doi:10.3390/antibiotics10040448)
Supplement: Supplementary file 1 [file antibiotics-10-00448-s001.zip › antibiotics-1165747-supplementary.pdf]

antibiotics

MDPI

Occurrence and Survival of Livestock-Associated MRSA in Pig Manure and on Agriculture Fields

Lærke Boye Astrup [1], Julie Elvekjær Hansen [1] and Karl Pedersen [2,*]

Table S1. Overview of sample types at different temperatures analyzed in the *in vitro* survival studies.

Sample types	5°C	15°C	25°C	37°C
Natural positive manure	A, B	C	-	A, B
Natural negative manure	D	D	D	D
Spiked manure	$D1^{+}$, $D2^{+}$<br>$D1^{++}$, $D2^{++}$	$D1^{+}$, $D2^{+}$<br>$D1^{++}$, $D2^{++}$	$D1^{+}$, $D2^{+}$<br>$D1^{++}$, $D2^{++}$	$D1^{+}$, $D2^{+}$<br>$D1^{++}$, $D2^{++}$

Three different natural positive samples = A, B and C from the same farm at different samplings.

Natural negative manure = D.

Natural negative manure spiked with ~ 10^3 CFU/mL MRSA t011 = $D1^{+}$ and MRSA t034 = $D2^{+}$.

Natural negative manure spiked with ~ 10^7 CFU/mL MRSA t011 = $D1^{++}$ and MRSA t034 = $D2^{++}$.
